# Medicinal plants sold at traditional markets in southern Ecuador

**DOI:** 10.1186/s13002-016-0100-4

**Published:** 2016-07-05

**Authors:** Fani Tinitana, Montserrat Rios, Juan Carlos Romero-Benavides, Marcelino de la Cruz Rot, Manuel Pardo-de-Santayana

**Affiliations:** Departamento de Ciencias Naturales, Universidad Técnica Particular de Loja, San Cayetano Alto, CP: 1101608 Loja, Ecuador; Departamento de Química, Universidad Técnica Particular de Loja, San Cayetano Alto, Loja, Ecuador; Institute for Tropical Ecology and Conservation, University of Florida, Gainesville, FL 32611-0430 USA; Área de Biodiversidad y Conservación, Departamento de Biología y Geología, Universidad Rey Juan Carlos, C/Tulipán s/n, 28933 Mostoles, Spain; Departamento de Biología (Botánica), Universidad Autónoma de Madrid, Campus de Canto Blanco, E-28049 Madrid, Spain

**Keywords:** Traditional markets, Medicinal plants, Factor Informant Consensus, Fidelity Level, Loja province, Southern Ecuador

## Abstract

**Background:**

The traditional markets in southern Ecuador and within the Andean region are especially important for plant resource trading among local people, even since before Spanish colonization; therefore, ethnobotanical studies are currently necessary and important. These strategic spaces persist for the traditional medicine cultural value reflected in the higher consumption of medicinal plants, which span all socioeconomic levels of rural and urban people. The purpose of this study includes the following: 1) to create a novel list of medicinal plants sold at 33 traditional markets; 2) to establish medicinal plant use agreement amongst vendors with the Factor of Informant Consensus (FIC); and 3) to determine the most sold medicinal plant species using the Fidelity Level (FL).

**Methods:**

This study focus on traditional markets ethnobotany utilizes the largest sample of medicinal plants market vendors up to date in Ecuador, interviewing them at 33 traditional markets, located within the Loja province. In order to determine the most sold medicinal plants and their ethnobotanical information, structured questionnaires and personal conversations were conducted with 196 medicinal plant vendors, and voucher specimens were created. Agreement among vendors about the therapeutic use of medicinal plants was measured using the FIC, and the most sold medicinal plant species were assessed with the FL.

**Results and discussion:**

This research registered 160 medicinal plant species, grouped in 126 genera and 57 families that were sold in 33 traditional markets. The uses of medicinal plants in southern Ecuador are related to a long history of traditional medicine health practices that has persisted until today as well as high plant diversity. The 53 therapeutic uses recorded were grouped into 12 medical categories that were adapted from the World Health Organization. Three medical categories shared the highest value for FIC = 0.92, which showed a high level of agreement of market vendors for 57 medicinal plant species sold to treat ailments related with digestive, dermatological, and sensorial systems. The FL index determined 11 culturally important medicinal plant species based on the reported uses by 40 or more market vendors. Two medicinal plant species had an FL = 100 %, *Matricaria recutita* and *Gaiadendrum punctatum*, used to treat digestive and respiratory systems ailments.

**Conclusions:**

In the Loja province, people continue to consume medicinal plant species sold at local markets to treat somatic and/or psychosomatic health ailments because sociocultural customs are strongly expressed in ancestral practices of wellbeing. When the largest values of FL (60.5 %–100 %) and FIC (0.81–0.92) indexes are combined, they demonstrated agreement among 196 market vendors in the use of seven medicinal plant species that were most sold for the 12 medical categories. This study stresses how important public policies are for the trade and quality of medicinal plant resources, particularly for local people practicing auto-medication. Reasons for the maintenance of traditional markets in southern Ecuador include lower cost of medicinal plants, confidence in traditional medicine, and/or sociocultural environment. In Ecuador, the sustainable management of wild medicinal plants diversity, particularly the most sold, is crucial for its conservation in nature.

## Background

Traditional markets around the world have been recognized as places for the trade of plants and their derivative products and have become exchange posts where cultures are expressed through regional trade [1–11]. Additionally, markets are a meeting place to display a diverse array of minerals, animals, and plants sold locally, which come from neighboring communities that are culturally and ecologically diverse [4, 12]. In this way, literature on traditional markets and traded medicinal plant species with their value chain flows requires more attention from scientists, because ethnobotanical information is rather scarce.

Current ethnobotanical research at traditional markets across continents, considering Asia, Africa, Oceania, and Latin America, contributes to the understanding of plant diversity through the trade of medicinal plant species and their cultural value [13–45]. In this way, market surveys can help to understand regional networks of producers, sellers, healers, and consumers by the supply and demand of medicinal plants and their derivative products [4]. The total number of inventoried medicinal plant species at a particular traditional market is important, but they do not necessarily represent all species used in the traditional medicine of a specific human group [5, 8].

According to the World Health Organization (WHO), the wellbeing of 80 % of the population in developing countries depends mostly on the use of medicinal plants through traditional medicine, spiritual therapies, and ancestral healing practices [46]. This fact is particularly evident in the ancestral practices of traditional communities living in rural areas. In Latin America, ethnobotanical studies of traditional markets and their history are needed, because the trade of medicinal plants and their derived products has local, national, regional, and international importance, especially given their growing demand [15, 47]. In the Andean-Amazonian region including Ecuador, traditional markets existed before Spanish colonization [48]. Throughout the Spanish conquest, a new kind of market appeared within public areas named “tiánguez” for the exchange of goods; they were also strategic points for bartering, conversation, and the sharing of life experiences [47, 48].

For the Andean region, published studies of traditional markets that emphasize ethnobotanical aspects have been conducted in Venezuela [11], Colombia [18], Bolivia [19, 43], Peru [49–79], and Ecuador [80]. Within Ecuador, it is estimated that 273 medicinal plants species were sold in the herb stalls (“puestos de hierbas”) of corresponding traditional markets, which were located at six provincial capitals in the Andean and Amazonian regions [80]. These capital cities, represented by Ambato, Quito, Riobamba, Nueva Loja, Puyo, and Tena, are the main points of trade for medicinal plants and their derivatives; from these places, commerce routes begin to spread throughout the country [81, 82].

More studies are needed to investigate the medicinal plants sold in Ecuadorian markets [80–85] to determine which medicinal plant species are most sold and how these are related to local health disorders. This is particularly true for the southern region of the country and specifically for the Loja province, because although it is a region rich in plant diversity, it is a region that is deficient in traditional market studies. In this area, only a few ethnobotanical surveys have been conducted, particularly on how the “mestizo” population and indigenous communities use medicinal plant resources from wild collection and/or homegardens [9, 52, 86–89].

Nowadays, even basic inventoried information accounting for the origins of medicinal plant resources and quantities of fresh and/or dry material sold is lacking as well as consumers’ usage of these products. Studies of traditional markets are necessary in Ecuador, because large gaps in knowledge on flora trade persist. This research at Loja province includes the following objectives: 1) to create a novel list of medicinal plants sold at 33 traditional markets; 2) to establish medicinal plants use agreement amongst vendors with the Factor of Informant Consensus (FIC) [90]; and 3) to determine the most sold medicinal plant species using the Fidelity Level (FL) [91].

## Methods

### Study area

**Fig. 1:**
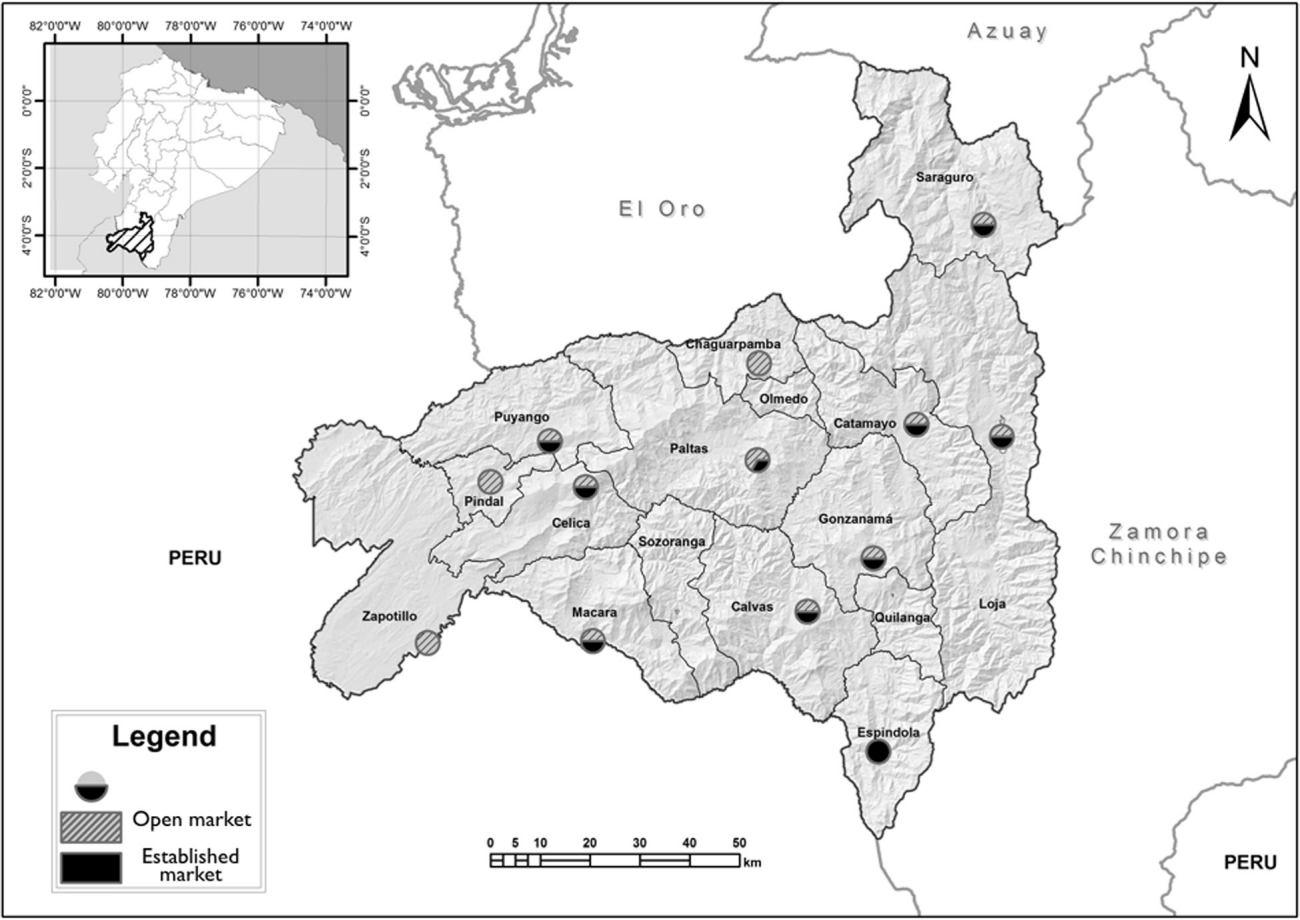
Location of 33 traditional markets within the Loja province, southern Ecuador

The study was carried out in 33 traditional markets within the Loja province, situated in southern Ecuador, between 3°19’56”S to 4°44’36”S latitude and 79°04’28”W to 80°29’03”W longitude (Fig. 1). This region occupies 11.042 km^2^, which is 4 % of the national territory, and borders to the south with Peru [92]. The total population of the province in 2010 was 448,966 inhabitants, consisting of 96.3 % “mestizo” Spanish speakers, and 3.7 % Saraguro indigenous people, who speak the Spanish and Kichwa languages [93].

The Loja province has abundant hydrographic features, like rivers that flow into the Pacific catchments basin. The province is dominated by the Andean mountain range, which gives rise to a very irregular topography, and altitudes between 120 and 3800 m. This region shows considerable variation in local climate, with conditions represented by tropical dry to the west, subtropical humid in the central area, and cold humid at the east [92].

### Markets

Medicinal plants were sold at 15 established markets and 18 open markets located in 13 of the 16 cities in Loja province (Fig. 1). The established market, known locally as “mercado”, includes from five to ten subsectors; the most frequent of these include the following: personal use items, electronic equipment, groceries, cooked foods, dairy products, meat products, legumes, fruits, and fresh or dry raw medicinal plants. Besides the main established markets, open markets occur weekly and are known locally as “feria libre”. Vendors at open markets are rural harvesters and/or small retailers who sell medicinal plants, fresh local products such as cheese, legumes, fruits, and vegetables; they commerce hens and guinea pigs.

A variety of actors were involved in the sale of medicinal plants at the studied markets. The majority of these actors included rural harvesters, small retailers, formal, and informal vendors (Table 1). The criteria applied to determine the types of each vendor in a market was based on how they auto-recognized their own role. The medicinal plant vendor in each market was essentially a person who used a specific know-how to trade bunches of medicinal plants, because each one is well familiarized with the therapeutic applications of every plant species sold. It is important to clarify that all interviewed vendors were just sellers and not healers.

**Table 1:** Kinds of vendors of medicinal plants and their role in traditional markets at Loja province

Vendor	Definition
Rural harvesters	Individuals who come from the rural areas surrounding the main cities of Loja province, bringing fresh medicinal plants produced and/or collected by them in nature and/or their homegardens. They always trade plant bunches in large quantities to formal vendors or small quantities to customers. They operate at open markets and/or established markets.
Small retailers	Individuals who come from rural areas surrounding the main cities of Loja province, bringing fresh medicinal plants harvested from nature or gathered from their homegardens. They occasionally go to the cities to trade plant bunches to the customers and/or to formal vendors in small quantities at open markets.
Formal vendors	Individuals who legally hold an operating license from the province government to rent a stall in the established market for trading legumes, fruits, vegetables and bunches of dry or fresh medicinal plants.
Informal vendors	Individuals who come from rural or metropolitan areas of Loja province, and are market vendors on foot. They are public resellers of fresh specific medicinal plant bunches in small quantities at established and/or open markets.

### Structured ethnobotanical questionnaires

Surveys of medicinal plants sold at 15 established markets and 18 open markets were conducted in the selected 13 cities within the Loja province between 2007 and 2013. During the visits, the first author carried out interviews with a total of 196 market vendors. After explaining the aim of the study, all vendors of medicinal plants from the 33 traditional markets were asked to participate in the research. The interviewed vendors were eighteen years or older; also, they were “mestizos” (95 %) and Saraguro indigenous people (5 %), and the large majority consisted of women (97 %).

Medicinal plants were bought from each vendor, and interviews were structured as ethnobotanical questionnaires in Spanish, being conducted by the main author with the 196 market vendors. In the field research, the first author respected the vendors who preferred to remain anonymous. The questionnaires aimed to record the specimens’ information on the following: vernacular names, medicinal uses, plant morphological structures sold, and therapeutic prescriptions. All the vendors who decided to collaborate were interviewed according to mutually agreed conditions and under Ecuador’s rights, especially with regards to the Convention on Biological Diversity (CDB) [94].

This research was conducted according to the code of ethics of the International Society for Ethnobiology (ISE) [95], which is also endorsed by the Society for Economic Botany (SEB). The Principle of Respect, numbered 9 in the code, recognizes the necessity for researchers to respect the integrity, morality, and spirituality of the culture, traditions, and relationships of indigenous people, traditional societies, and local communities within their worlds.

### Voucher collection and nomenclature

The nomenclature of plant families, genera, and species follows the Catalogue of Vascular Plants of Ecuador [96]. It was also compared to the TROPICOS database [97]. The 160 species were identified using the available volumes of the Flora of Ecuador [98–101] and reference material in the herbaria of the “Universidad Técnica Particular de Loja” (HUTPL) and “Universidad Nacional de Loja” (LOJA). The specimens were registered under the collection series FT (Fani Tinitana), and vouchers were deposited at HUTPL. The collection of botanical specimens sold in the 33 traditional markets authorized the Ecuadorian Ministry of the Environment (Ministerio del Ambiente del Ecuador N° 001-2013-IC-FLO-DPAP-MAE).

### Quantitative analysis

All the local therapeutic uses of medicinal plants were grouped in 12 medical categories (Table 2), which were adapted from the catalogue of International Classification of Diseases made by the WHO [102]. In this research, each category proposed by the WHO allows grouping and systematizing the data related to ‘illness’ and ‘disease’ as well as to compare the results among other regional and international studies related to the markets’ ethnobotany [102, 103]. Additionally, WHO recognizes in each medical category the health practice systems of traditional populations [46]. In this study, ‘illness’ refers to being ill as conceived from a sociocultural personal perception, while ‘disease’ was considered from the biomedical perspective [104].

**Table 2:** Therapeutic uses of medicinal plants to treat local ailments at Loja province

Medical category	Local illnesses and diseases recognized by market vendors
Circulatory system	Anemia, bad blood circulation, high cholesterol, and high or low blood pressure
Culture-bound syndromes	“Calor encerrado”, evil air, evil eye, fright, “espanto de cerro”, and “pena de ausencia” or “tirisia” (see definition of syndromes in Tene et al. 2007) [88] and Rios et al. 2007 [104]
Dermatological system	Acne, fungus infection, gangrene, rash, wounds, nosebleed, hair loss, and dandruff
Digestive system	Diarrhea, constipation, sickness, hangover, flatulence, liver disorder (included inflammation), stomach infection and pain, and tooth pain
General disorders	Inflammation, cancer, fever, headache, and sunstroke
Genitourinary system	Kidney ailments (included inflammation and infection), and prostate and urinary tract disorders
Gynecological system	Vaginal disorders, abdominal pain, menstrual cramps and related disorders, ovary inflammation, and promoting labor and childbirth recovery
Hormonal system	Diabetes and galactogogue
Musculoskeletal system	Bone fracture, bruise, rheumatism, sprain, and pains
Nervous system	Nervousness
Respiratory system	Cold, cough, flu, sore throat, and measles
Sensorial system	Ear pain and eye infection

Information recorded in the structured ethnobotanical questionnaires related to the collected 160 taxa and their medicinal uses were recorded into a data matrix for quantitative analysis. The FIC index was used to measure consensus among vendors regarding the therapeutic use of each medicinal plant [106–111]; it shows the level of homogeneity among information provided by different vendors. The FIC was calculated according to the following formula: FIC = (N_ur_ – N_t_)/(N_ur_ – 1), where N_ur_ refers to the number of therapeutic use reports, grouped in a medical category, from market vendors for a particular medicinal plant, and N_t_ refers to the total number of medicinal plant species used in a particular medical category [90, 106, 107]. The FIC values range between 0 and 1, where 1 indicates the highest level of market vendor consensus.

The relative healing potential of each reported medicinal plant sold at 33 traditional markets was evaluated with the FL index [91–112]. This indicates the percentage of vendors claiming the use of a certain medicinal plant for the same therapeutic use, which was grouped in a specific medical category [113, 114]. The FL was calculated according to the following formula: FL (%) = (I_p_ × 100/I_u_), where I_p_ is the number of market vendors who independently claim a therapeutic use of a medicinal plant species to treat a specific illness or disease, and I_u_ is the total number of market vendors that sold the same medicinal plant to treat any given illness or disease.

## Results and discussion

### Medicinal plants sold at traditional markets

This research registered 160 medicinal plant species traded in 33 traditional markets within the Loja province, which were grouped in 123 genera and 57 vascular plant families (Table 5.). In traditional markets at La Paz (Bolivia) and Cusco (Peru), a total of 129 [19] and 152 [117] medicinal plant species were respectively reported; in contrast, 400 plant species were recorded in Trujillo and Chiclayo (Peru) [22]. When compared to these previous studies, the number of medicinal plant species sold in markets within the Loja province represents an intermediate value.

**Fig. 2:**
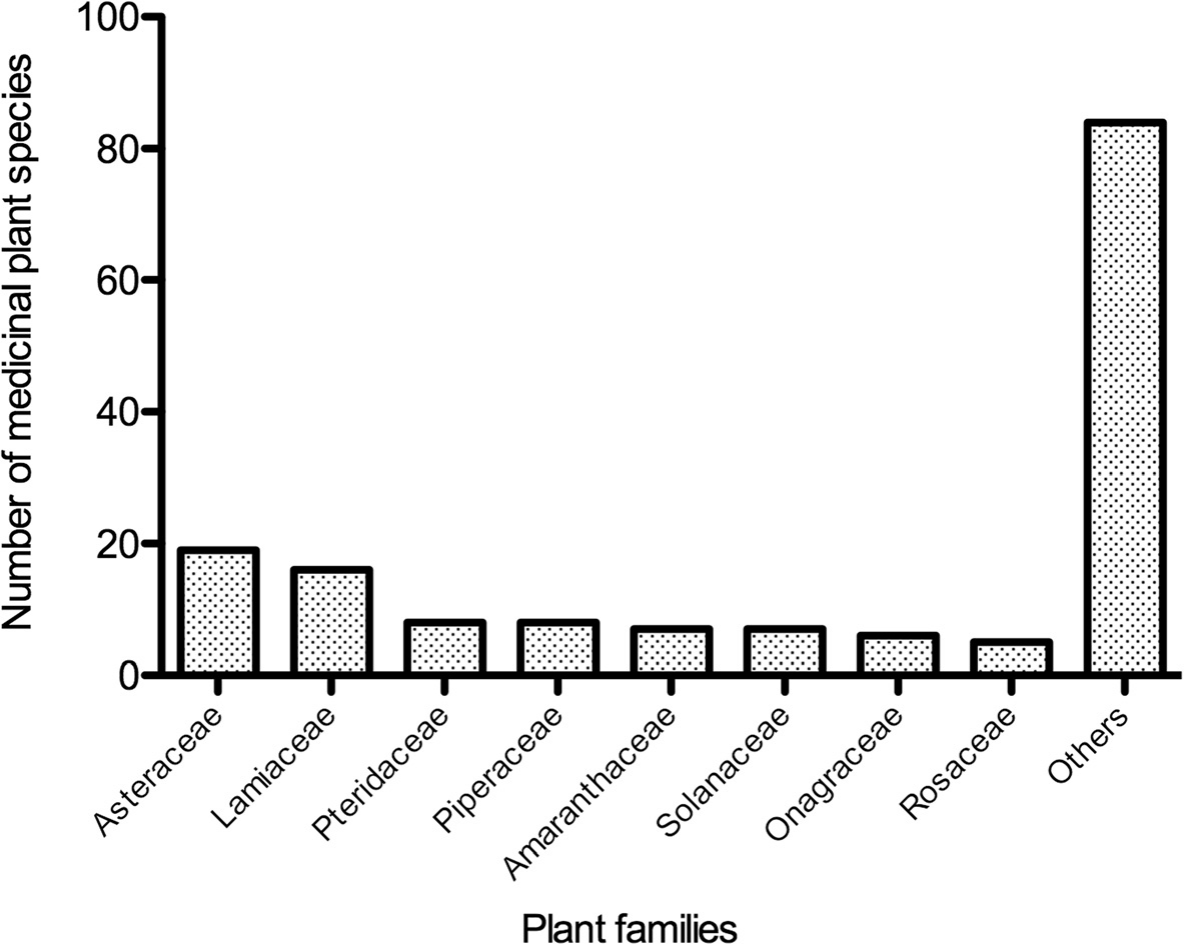
Dominant medicinal plant families recorded in 33 traditional markets within the Loja province, southern Ecuador

The dominant plant family was Asteraceae with 19 species that represented 11.8 % of the total species, followed by 16 species of Lamiaceae (10 %), 8 species of Piperaceae (5 %) and Pteridacea (5 %), 7 species of Amaranthaceae (4.4 %) and Solanaceae (4.4 %), 6 species of Onagraceae (3.8 %), and 5 species of Rosaceae (3.1 %) (Fig. 2). Other studies of Andean highland traditional markets also recorded Asteraceae as the family with the highest number of medicinal plant species, and Solanaceae and Lamiaceae were consistently among the most frequent families [11, 43, 117].

The most frequent medicinal plant life forms were herbs (61.1 %) and shrubs (32.2 %), followed by trees (5.5 %) and lianas (1.2 %). This data was similar to results from other studies of highland markets in Bolivia [19] and Peru [117], where the herb habit represents a large percentage due to its random occurrence, high diversity, and endemism. Herbs such as weeds, which were abundantly available in relation to other plant life forms, are an important source of food and remedies [118]. This is because they contain one or more bioactive principles and a wide variety of highly active secondary metabolic compounds [119], making these plants potentially more effective for medicinal applications [120].

### Geographic status of medicinal plant species sold at traditional markets

The medicinal plants native to Ecuador belonged to 92 species (57.5 %) [82, 86, 88] and were brought to traditional markets from mountain forests, cloud forests, scrub vegetation, and the Andean paramo [121, 122]. Of the 160 species, 6 (3.8 %) were endemic to Ecuador highlands [123], corresponding to the families Asclepiadaceae (*Orthosia ellemanniae*), Asteraceae (*Achyro clinehallii, Aequatorium jamesonii* and *Aristeguietia persicifolia*), and Onagraceae (*Fuchsia harlingii* and *Fuchsia loxensis*), whereas 62 species (38.7 %) were introduced from different regions of the world. In relation to the role of humans in the management of medicinal plant species, the material sold in the studied traditional markets belonged to homegardens (cultivation) and natural vegetation (wild).

The five most sold medicinal plant species were very well known by vendors for their therapeutic uses and health properties. These were *Aerva saguinolenta*, *Equisetum bogotense*, *Matricaria recutita*, *Oreocallis grandiflora*, and *Ruta graveolens*. The trade of these five taxa was linked to the treatment of the most common illnesses and diseases present within the Loja province, related to the respiratory system, genitourinary system, digestive system, and culture-bound syndromes (e.g. “evil eye”, “evil air”, “frights”, and “calor encerrado”). It is important to stress that *Matricaria recutita* and *Ruta graveolens* are widely traded and used throughout South America and the Old World [19, 62, 124], because of their magical and medicinal qualities for the preparation of remedies used in therapies of soul and body.

The five plant species most commonly used in medicinal beverages, locally known as “horchata” (herbal mixture tea) and “agua aromática” (herbal tea), were native and cultivated [96]. These were *Aloysia triphylla, Amaranthus hybridus*, *Pelargonium graveolens*, *Equisetum bogotense*, and *Oreocallis grandiflora*. The availability of these plant species was firstly revealed by their spatial accessibility, explained by their wide spread distribution and cultivation; secondly, it was explicated by their seasonal stock and the quantities of plant material sold throughout the year. Similarly to Venezuela, Peru, and Bolivia, these plant species are used individually or in herbal mixtures infusions [11, 19, 65], especially to treat different kinds of afflictions related to the nervous, digestive, and genitourinary systems.

### Vernacular names of medicinal plant species traded in Loja province

A total of 204 vernacular names were recorded for the 160 medicinal plant species; 59.4 % of the species had at least one name, 36.1 % had two names and 4.5 % had three names. Most of the vernacular names used to identify each plant were in Spanish, followed by a few in Kichwa language from the Andean highlands. The name given to the whole plant is the same name given to the plant’s morphological structure. In the case of gathering wild plant species belonging to the *Malva* genus or cultivated hybrids of the *Fuchsia* genus, the vendors recognized all the species in the genus with the same vernacular name.

The vernacular names of the medicinal plants compiled in this study were compared to others from previous studies conducted at other traditional markets in Bolivia [19], Peru [22, 77], and Ecuador [83] as well as ethnobotanical surveys in the Andean highlands [125] and Spain [124]. The most common five medicinal plant species that share the same cosmopolitan vernacular names were *Dysphania ambrosioides* (“paico”), *Equisetum giganteum* (“cola de caballo”), *Matricaria recutita* (“manzanilla”), *Melissa officinalis* (“toronjil”), and *Rosmarinus officinalis* (“romero”).

### Medicinal plants: morphological structures and therapeutic administration

The therapeutic administration recommended by the market vendors revealed the 13 kinds of medicinal plant morphological structures sold, where each one was used to treat a human organ and/or fluid. The most frequently traded morphological structures were branches for 44 medicinal plant species (27.5 %), followed by leaves (25.6 %), flowers (16.9 %), and plants without roots (16.9 %). The less frequently sold morphological structures, available only at 18 open markets, were bark, fruit, inflorescence, latex, seed, stem, style, root, and wood.

The research identified 20 modes of therapeutic administration (Table 5): 14 were prepared using fresh plant material sold at 18 open markets, and six used dried plant material sold at 15 established markets. Oral was the most frequent mode of therapeutic administration (83.8 %), prepared with fresh and/or dry plants, especially in a drink locally known as “bajeada”. Compared to other Andean markets, oral infusions have similar preparations in Bolivia [19] and Cuzco [117].

Other therapeutic administrations were rubbing (16.9 %), topical applications (8.7 %), hot baths after childbirth (5.6 %), and cleaning wounds (5 %). The majority of the prescribed medicinal plants given to patients by vendors were applied without any standardized doses. Only a few elder vendors made warnings about adverse side effects of some medicinal plants, but they never mentioned antidotes. The six most common orally administered medicinal plant species were *Aerva sanguinolenta*, *Amaranthus hybridus*, *Equisetum bogotense, Matricaria recutita*, *Melissa officinalis,* and *Oreocallis grandiflora.*

The therapeutic administration, locally known with the term “zumo”, generally refers to the extract of a plant morphological structure or fruit pure juice, thus differentiating “zumo” from “jugo” (juice); the latter is associated with the fruit diluted in an amount of water [104]. The five most popular medicinal plant species sold for “zumo” were *Aerva sanguinolenta* (plant without root)*, Cardamine bonariensis* (plant without root), *Peperomia blanda* (plant without root), *Tradescantia zebrina* (leave), and *Verbena litoralis* (branch).

### Factor of Informant Consensus (FIC)

In studies related to medicinal plants, the FIC index provides a measure of reliability for the specified statement of evidence regarding the agreement amongst a specific human group [90, 126]. The 160 medicinal plants sold in 33 traditional markets to treat different human ailments were classified into 12 medical categories, with a FIC value assigned to each (Table 3). Three medical categories shared the highest value for FIC = 0.92, which showed a high level of agreement amongst the 196 vendors for 57 medicinal plant species sold to treat the digestive, dermatological, and sensorial systems.

**Table 3:** Medical categories and Factor of Informant Consensus among vendors of traditional markets at Loja province

N^o^	Medical category^a^	Number of medicinal plant species	Percentage of all medicinal plant species	Use citations bymarket vendor	Percentage of all use citations	FIC^b^
1	Digestive system	37	21.89	437	12.31	0.92
2	Dermatological system	16	9.47	186	5.24	0.92
3	Sensorial system	4	2.37	39	1.10	0.92
4	Culture-bound syndromes	34	20.12	359	10.11	0.91
5	Nervous system	20	11.83	215	6.05	0.91
6	Respiratory system	29	17.16	321	9.04	0.91
7	Genitourinary system	29	17.16	313	8.81	0.91
8	Circulatory system	16	9.47	123	3.46	0.88
9	General disorders	46	27.22	352	9.91	0.87
10	Gynecological system	28	16.57	180	5.07	0.85
11	Musculoskeletal system	13	7.69	71	2.00	0.83
12	Hormonal system	7	4.14	33	0.93	0.81

^a^The medical categories were adapted from International Classification of Diseases catalogue provided by World Health Organization and applied to group 160 medicinal plant species sold at 33 traditional markets within the Loja province, southern Ecuador

^b^ *FIC* Factor of Informant Consensus

The digestive system has the highest value of used citations by 437 market vendors, who report 37 medicinal plant species. This is related to a high incidence of gastrointestinal ailments in southern Ecuador [86, 127], which may also be associated to stomach cancer, as this is the second most important cause of mortality in the country [128]. When comparing the large FIC values obtained in this study for the digestive system to the most important medical categories reported in previous surveys worldwide [113, 115, 116, 129], the results reveal the importance of medicinal plant species in treating ailments of the digestive system, such as gastric complaints and abdominal pains.

Additionally, therapeutic uses related with the other two medical categories, culture-bound syndromes and general disorders, had relatively high values of use citations by market vendors. The medicinal plant species responsible for this particular status was *Ruta graveolens*, used to treat seven psychosomatic complaints, and *Equisetum bogotense,* used in five common ailments. As it was confirmed by participative observation, local people believe that these two medicinal plant species were efficient in the treatment of 12 particular local ailments, revealing the persistence of cultural believes syndromes and the necessity of preventive medicine to avoid common ailments.

Even the lowest FIC values registered in this study, which included hormonal system (0.81), musculoskeletal system (0.83), and gynecological system (0.85), are large when compared to other studies that use this same index [106, 107, 110, 111, 130]. These low FIC values show a low agreement amongst medicinal plant market vendors, specifically in the trade of medicinal plant species associated to the treatment of the symptoms related to these ailments. This data contrasts with the results of a market study in Venezuela [30], where a FIC = 0.91 was registered for the gynecological system, as obtained with 25 interviewed market vendors.

The FIC values between 0.81 and 0.92 demonstrate the strong levels of consensus amongst 196 vendors in the multiple uses of the 160 medicinal plant species sold. Incidentally, even when the taxa number prescribed for a specific illness or disease varied, the majority of the 33 traditional markets had a common pool of regional flora sold to clients within the 12 medical categories. As in Bolivia [19] and Peru [125], the most common medicinal plant species sold were for symptoms related to the digestive system, nervous system, respiratory system, and genitourinary system.

### Fidelity Level (FL)

The FL index determined 11 culturally important medicinal plant species in the local population of the Loja province (Table 4), as based on the reported uses by 40 or more market vendors to treat 53 illnesses and diseases grouped in 12 medical categories. It was also useful for highlighting the most important species sold in each medical category. In this analysis, the 160 medicinal plant species mentioned by market vendors were considered, and the FL was calculated for each one. A FL of 100 % for a specific medicinal plant species indicates that all of the plant use reports mentioned the same therapeutic administrations to treat an illness or disease.

**Table 4:** Most used medicinal plants species for medical categories based on highest fidelity level at Loja province

N^o^	Medicinal plant species	Medical category^a^	I_p_	I_u_	FL value (%)^b^
1	*Matricaria recutita* L.	Digestive system	57	57	100
2	*Gaiadendron punctatum* (Ruiz & Pav.) G. Don	Respiratory system	44	44	100
3	*Ruta graveolens* L.	Culture-bound syndromes	41	46	89.1
4	*Melissa officinalis* L.	Nervous system	58	85	68.2
5	*Equisetum bogotense* Kunth	General disorders	78	116	67.2
6	*Amaranthus hybridus* L.	Circulatory system	47	73	64.4
7	*Viola tricolor* L.	Dermatological system	46	76	60.5
8	*Borago officinalis* L.	Respiratory system	49	120	40.8
9	*Oreocallis grandiflora* (Lam.) R. Br.	Genitourinary system	40	107	37.4
10	*Sambucus nigra* L.	Respiratory system	65	186	34.9
11	*Aerva sanguinolenta* (L.) Blume	Gynecological system	78	238	32.8

^a^The medical categories were adapted from International Classification of Diseases catalogue provided by World Health Organization and applied to 160 medicinal plant species sold at 33 traditional markets within the Loja province, southern Ecuador

I_p_ = Number of market vendors who independently cited the importance of a specific illness or disease

I_u_ = Total number of market vendors

^b^FL value % = Fidelity Level value percentage (0 = the least, 100 = the highest efficiency)

Two medicinal plant species had a FL = 100 %, *Matricaria recutita* and *Gaiadendrum punctatum*, because they were used consistently for ailments in the digestive system and respiratory system, respectively. This may be due to their greater efficacy in alleviating symptoms and the persistence of ancestral wisdom beliefs in the local population. These two species have considerable agreement amongst market vendors on their particular use and credibility and therefore could be further analyzed for developing pharmafood or pharmaceutical products.

The market vendors had a tendency to rely on 11 medicinal plant species to treat ailments related to nine medical categories. The most important seven had an FL > 60 % and represented *Matricaria recutita, Gaiadendron punctatum, Ruta graveolens, Melissa officinalis, Equisetum bogotense, Amaranthus hybridus,* and *Viola tricolor*. All these medicinal plant species should be studied to determine the efficacy and safety of all local reported medical uses and also evaluated by phytochemical and pharmacological tests as well as bioactivity essays and toxicity studies.

## Conclusions

Within the Loja province, people continue to use traditional medicine by consuming particular medicinal plant species sold at local markets. This also shows that sociocultural customs are strongly expressed in ancestral practices of wellbeing. Proof of the former was found by analyzing responses from 196 vendors who sold 160 fresh or dried medicinal plant species material to treat a wide spectrum of illnesses and diseases. The plant resources functioned as palliatives or, in some cases, curatives to both somatic and psychosomatic health afflictions. Nowadays in Ecuador, all stake-holders related with dynamics of traditional markets networks (e.g., all kinds of vendors and local people), who are frequently using medicinal plants require detailed research for a safety and serious use.

In the case of culture-bound syndromes plus complications with a diversity of systems such as digestive, sensorial, dermatological, respiratory, genitourinary and/or nervous, there are usually no precise therapeutical prescriptions. The separation between illnesses and diseases is very small, especially with regards to what the therapeutic administration and medical treatment should be. An example is the *Ruta graveolens* based-remedy, which is used to relieve “evil eye”, “evil air”, “frights”, and “calor encerrado” plus menstrual disorders.

The agreement among 196 market vendors in the use of seven specific most sold medicinal plant species for the 12 medical categories is fairly high, especially when the largest values of FL (60.5 %–100 %) and FIC (0.81–0.92) indexes are combined. A total of seven plant species with a high FL value for treating gastro-intestinal diseases are under investigation for their pharmacological properties by the Applied Chemical Department of the UTPL research team.

For future efforts, it should be important to focus on correlating the values of FL and FIC with the incidence of local ailments, as this will be useful to establish public health policies related with the trade of medicinal plant species. This initiative will be effective to support traditional medicine and its therapeutic repertoire. The first step will be to choose the medicinal plant species with widespread and consistent medicinal use in southern Ecuador and to study their therapeutical applications with physicians and scientists, primarily to identify bioactive compounds.

The evidence presented in this study reaffirms the relationship between ancestral wisdom and traditional medicine, particularly in local markets within the Loja province. In fact, it is important to stress how medicinal plant resources are crucial for local people in 13 cities within the Loja province; also, it is important to understand why a high percentage of them practice auto-medication. Reasons for the maintenance of traditional markets include lower cost of plant products, confidence in traditional medicine, and/or sociocultural environment.

This research is the first contribution to understanding from the ethnobotanical point of view the human-plant dynamics of traditional markets within the Loja province, where medicinal plants have a substantial role in the lives of local people. The trade demand of medicinal plants and their derivatives over the next few years could increase, leading to the over-harvesting of wild plant species and could perhaps even endanger natural populations, (e.g., *Oreocallis grandiflora*). Sustainable management of wild medicinal plants is important for their diversity conservation and in order to avoid their extinction, particularly in the case of highly used species in traditional medicine.
